# A comparison of mRNA and circRNA expression between squamous cell carcinoma and adenocarcinoma of the lungs

**DOI:** 10.1590/1678-4685-GMB-2020-0054

**Published:** 2020-11-06

**Authors:** Min Yu, Yingxuan Tian, Min Wu, Jinglong Gao, Yuan Wang, Fuqiang Liu, Sen Sheng, Shufen Huo, Jun Bai

**Affiliations:** 1 Shaanxi Provincial People's Hospital Shaanxi Provincial People's Hospital Department of Oncology Medicine Xi'anShaanxi China Shaanxi Provincial People's Hospital, Department of Oncology Medicine, Xi'an, Shaanxi, China.; 2 Shaanxi Provincial People's Hospital Shaanxi Provincial People's Hospital Department of Elderly Respiratory Medicine Xi'anShaanxi China Shaanxi Provincial People's Hospital, Department of Elderly Respiratory Medicine, Xi'an, Shaanxi, China.; 3 Shaanxi Provincial People's Hospital Shaanxi Provincial People's Hospital Xi'an Medical College Xi'anShaanxi China Shaanxi Provincial People's Hospital, Xi'an Medical College, Department of Elderly Medicine, Xi'an, Shaanxi, China.; 4 Shaanxi Provincial People's Hospital Shaanxi Provincial People's Hospital Department of Elderly Medicine Xi'anShaanxi China Shaanxi Provincial People's Hospital, Department of Elderly Medicine, Xi'an, Shaanxi, China.; 5 Universidade Federal do Rio Grande do Sul Universidade Federal do Rio Grande do Sul Shaanxi Provincial People's Hospital Department of Preventive Health Section Xi'anShaanxi China Shaanxi Provincial People's Hospital, Department of Preventive Health Section, Xi'an, Shaanxi, China.; 6 Shaanxi Provincial People's Hospital Shaanxi Provincial People's Hospital Cardiovascular Department Xi'anShaanxi China Shaanxi Provincial People's Hospital, Cardiovascular Department, Xi'an, Shaanxi, China.; 7 University of Arkansas for Medical Science University of Arkansas for Medical Science Neurology Department Little RockAR USA University of Arkansas for Medical Science, Neurology Department, Little Rock, AR, USA.

**Keywords:** Non–small-cell lung cancer, circRNAs, lung adenocarcinoma, lung squamous cell carcinoma, ceRNA network, survival analysis

## Abstract

Lung squamous cell carcinoma (LUSC) and lung adenocarcinoma (LUAD) are the two major subtypes of non–small-cell lung cancer (NSCLC). This study aimed to compare mRNA and circRNA expression patterns between LUSC and LUAD. Cancer tissues from 8 LUSC patients and 12 LUAD patients were collected to obtain mRNA and circRNA expression profiles. The differentially expressed mRNAs (DEmRNAs) and circRNAs (DE-circRNAs) between LUSC and LUAD were screened. Afterwards, miRNA–DEcircRNA pairs and miRNA–DEmRNA pairs were predicted to construct a competing endogenous RNAs (ceRNAs) network, followed by functional enrichment analysis and survival analysis. In total, 635 DEmRNAs and 245 DEcircRNAs were obtained. The ceRNA analysis revealed that genes, such as *EPHA2*, *EPHA7*, *NTRK2*, *CDK6*, hsa_circ_027570, hsa_circ_006089, and hsa-circ_035997, had distinct expression patterns between LUSC and LUAD. Also, functional enrichment analysis indicated that DEmRNAs were mainly enriched in *ERK1* and *ERK2* cascade. Survival analyses suggested that *STXBP1* and *PMEPA1* were associated the prognosis of with both LUAD and LUSC, whereas *EPHA2* and *CDK6* might serve as prognostic factors for LUSC and LUAD, respectively. In conclusion, genes such as *EPHA2*, *EPHA7*, *NTRK2*, and *CDK6* had different patterns in the two major histological subtypes of NSCLC. Notably, *EPHA2* and *CDK6* might be considered as potential therapeutic targets for LUSC and LUAD, respectively.

## Introduction

Non–small-cell lung cancer (NSCLC) is a prevalent malignant tumor characterized by a considerably high incidence and mortality (Jacky and Baik, 2017). Despite some achievements in NSCLC treatment, the overall survival is still poor (Dreyer *et al.*, 2018). Recently, with the identification of oncogenes and development of corresponding targeted therapies, molecular testing has become an important means for treating NSCLC (Nellesen *et al.*, 2018). Thus, understanding the molecular basis of NSCLC progression is vital to improve the treatment and prognosis of patients. The most abundant subtypes of NSCLC are lung squamous cell carcinoma (LUSC), and lung adenocarcinoma (LUAD), which are distinct in their histological, molecular, and clinical characteristics; thus, accurate classification of NSCLC into LUAD and LUSC is essential for both clinical practice and lung cancer research (Coudray *et al.*, 2018).

Accumulating evidence has reported the similarities and differences of gene expression patterns between LUAD and LUSC. Yang *et al.* (2019) investigated the differences in the gene expression and methylation patterns of LUAD and LUSC, and observed that cathepsin E (*CTSE*) and solute carrier family 5 member 7 (*SLC5A7*) could be considered as potential biomarkers in the personalized treatment for LUAD and LUSC. Liu *et al.* (2017) found that lncRNA DGCR5 was correlated with the prognosis of LUSC, whereas MIR31HG was associated with the overall survival of LUAD, indicating that these lncRNAs had diagnostic and therapeutic potential in for LUSC and LUAD treatment. Furthermore, Wang *et al.* (2019) demonstrated that hsa_circ_0001073 and hsa_circ_0001495 as diagnostic markers for LUAD and LUSC, respectively. Despite the above studies, the differences of mRNA and circRNA in two major subtypes of NSCLC have not been fully explored.

This study aimed to explore the differences in the gene expression of LUAD and LUSC by comparing the mRNA and circRNA expression profiles. In addition, the prognostic genes related to LUSC and LUAD were also respectively identified. Our study could provide more insights into the molecular mechanism of LUSC and LUAD, and provide potential prognostic and diagnostic biomarkers for NSCLC.

## Material and Methods

### Sample collection

All lung cancer tissues were collected from 20 patients, including 8 LUSC and 12 LUAD, who were admitted to Shanxi Provincial People's Hospital, China between October 2015 and March 2018. Histopathological diagnosis of all specimens was in accordance with the World Health Organization criteria for pathological classification of lung cancer and AJCC/UICC staging system for lung cancer. Furthermore, surgical resection complied with the standards from NCCN guideline staging and surgical indications. The excluding criteria of samples were as follows: 1) patients who refuse surgery; 2) patients who do not undergo pulmonary lobectomy and receive general anesthesia; 3) patients with contraindications to anesthesia; and 4) patients who have received adjuvant therapy. The clinical characteristics of included patients are shown in Table S1. This study was approved by the Ethics Committee for Shanxi Provincial People's Hospital and the University of Arkansas for Medical Science. Informed consent was obtained.

### RNA extraction and sequencing

To meet the sequencing criteria, each sample required a certain amount of repeated measurement data. Therefore, we created three repetitions for each group. Three LUAD groups and three LUSC groups were then subjected to transcriptome sequencing. Total RNA of the collected samples was extracted using TRIzol kit following the manufacturer's procedure. The concentration and purity of RNA were detected using a NanoPhotometerN60 (IMPLEN, Munich, Germany). The integrity of RNA was examined using the Agilent 2100 Bioanalyzer (Agilent Corporation, CA, USA). Then, rRNA from the total RNA was depleted using Ribo-Zero GoldrRNARemova Kit (Illumina, San Diego, USA). The treated RNAs were interrupted randomly to 200–300 bp to construct cDNA library. The insert size and total concentration of library were detected using Agilent 2100 Bioanalyzer and qPCR. Finally, high-quality library was sequenced on the Illumina HiSeq platform.

### Data processing and identification of RNAs

To ensure the accuracy of the information analysis, the quality control of the sequencing data was performed to obtain clean reads. Reference genome was acquired from Ensembl database (http://www.ensembl.org/org/). Then, the clean data were aligned to the reference genome to obtain the mapped data.

HTseq 0.6.1p2 (http://www huber.embl.de/users/anders/HTSeq) was applied to calculate the read count value of each mRNA and find_circ was used to identify the counts of circRNA. The raw counts of mRNA and circRNA were normalized using reads per kilo bases per million reads (RPKM).

### Identification of differentially expressed mRNAs and circRNAs

After sequencing, raw expression levels were processed by log_2_ transformation. Differential expression analysis between LUSC and LUAD was performed using a paired *t-*test with Benjamini–Hoachberg method. The log_2_FC (fold change) > 1 and adjusted *p* value (adj. *p* value) < 0.05 were set as cut-off threshold of DEmRNA and DEcircRNA. The heatmap of DE-mRNAs and DE-circRNAs were constructed using pheatmap (version: 1.0.10, https://cran.r-project.org/web/packages/pheatmap/index.html) software in R.

### Construction of miRNA regulatory network

We adopted the overrepresentation analysis (ORA) method in WebGestalt (http://www.webgestalt.org/option.php) (Zhang *et al.*, 2005) to predict miRNAs associated with DEmRNAs. The *p* value < 0.05 was set as the significant level. Afterwards, miRNA–DEmRNA pairs were identified and visualized using Cytoscape software. In addition, miRanda (version 3.3a, https://omictools.com/miranda-tool) (Agarwal *et al.*, 2018) was utilized to predict potential target miRNAs of the DEcircRNAs. The miRNA–DEcircRNA pairs with score > 150 and energy < −20 were selected to establish a regulatory network.

### CeRNA network construction

The ceRNA network was established to explore and determine the interactive relationship between mRNAs, miRNAs, and circRNAs. Notably, mRNAs–DEcircRNAs positive correlation pairs were further extracted with the cutoffs of *p* value < 0.05 and r > 0.9. Following this, DEcircRNA–miRNA–DEmRNA ceRNA network was structured by combining miRNAs–DEmRNAs pairs and DEmRNAs–DEcircRNAs positive correlation pairs.

### Functional enrichment analysis of DEmRNAs

To better understand the biological functions of genes, the functional enrichment analysis of DEmRNAs existed in ceRNA network was performed using DAVID (version 6.8, https://david-d.ncifcrf.gov/) (Huang *et al.*, 2009). GO-biological process (BP) terms (Ashburner *et al.*, 2000) or pathways (Kanehisa and Goto, 2000) with the threshold of *p* < 0.05 and gene count > 2 were considered as statistically significant.

### Survival analysis

TCGA database provides various expression profiling and clinical data, which contributes to improve the accuracy of prevention and diagnosis of various cancers (Tomczak *et al.*, 2015). The gene expression profiles and clinical information of LUAD and LUSC were obtained from TCGA database, including 501 LUSC samples and 517 LUAD samples. DEmRNAs in ceRNA network were divided into high expression group and low expression group according to the median of expression values. The Kaplan–Meier curves were acquired using survival package (version: 2.42-6, https://cran.r-project.org/web/packages/survival/index.html) in R. The *p* value < 0.05 was considered statistically significant.

## Results

### RNA sequencing overview

We obtained 427,794,228 raw data from LUSC samples and 412,360,880 raw data from LUAD samples. The LUSC and LUAD clean reads accounted for more than 99.5% and 99.6% of raw data, suggesting that our data were high quality and could be used for subsequent analysis. Ultimately, a total of 17284 mRNAs and 4997 circRNAs were screened.

### Identification of DEmRNAs and DEcircRNAs

**Figure 1:**
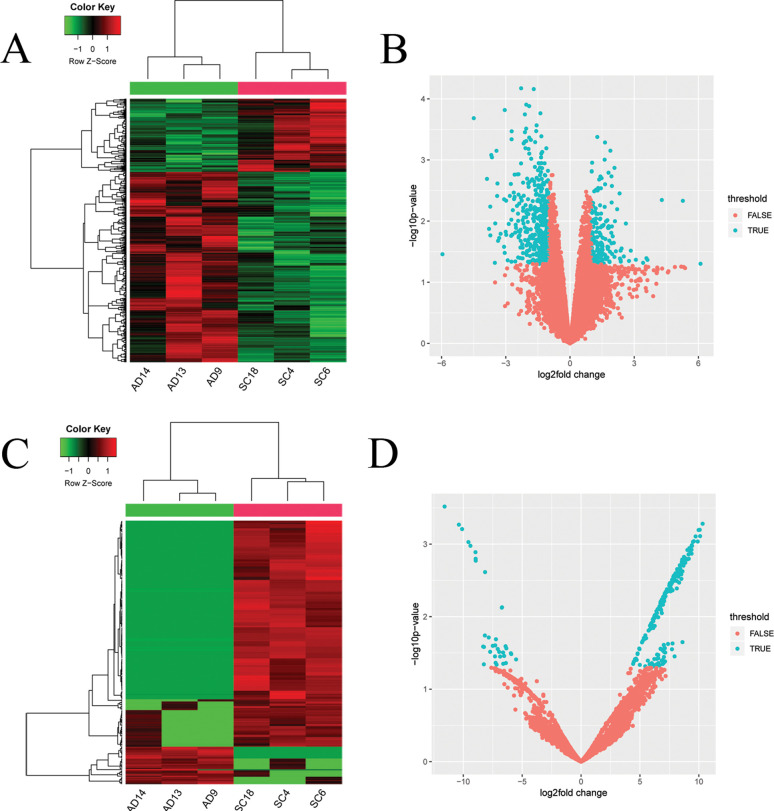
Heatmaps and volcano plots of DEmiRNAs differentially expressed mRNAs and DEcircRNA, miRNAs and lncRNAs. (A) heatmap for DEmRNAs, (B) volcano plot for DEmRNAs, (C) heatmap for DEcircRNA, and (D) heatmap for DEcircRNA. DEmRNAs: differentially expressed mRNAs; DEcircRNA: differentially expressed circRNAs.

A total of 635 DE-mRNAs (176 up- and 459 down-regulated) and 245 DEcircRNA (210 up- and 35 down-regulated) were obtained in LUAD vs LUSC. The complete list of DEmRNAs and DEcircRNAs is shown in Table S2 and Table S3. Hierarchical cluster analyses on DEmRNAs (Figure 1A) and DEcircRNAs (Figure 1C) indicated that LUSC clearly segregated from LUAD. In addition, the volcano diagrams of DEmRNAs (Figure 1B) and DEcircRNAs (Figure 1D) are displayed.

### *Construction of miRNA-mRNA and circRNA-miRNA networks*

**Figure 2:**
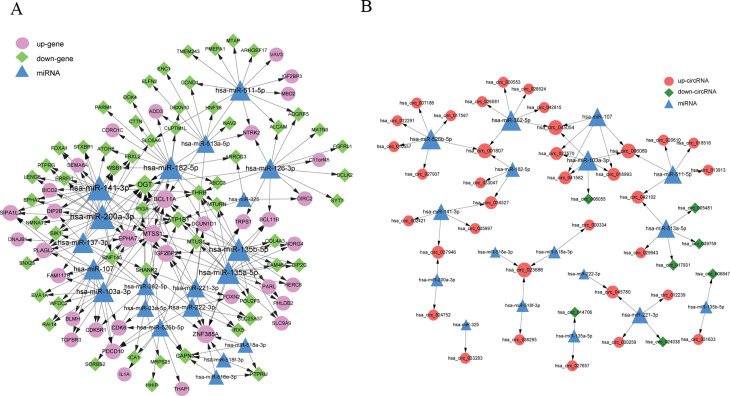
The miRNA–mRNA and miRNA–circRNA regulatory networks. (A) miRNA–mRNA network and (B) miRNA–circRNA regulatory network. Blue triangles indicate miRNA, pink circles represent down-regulated genes, light green rhombuses indicate down-regulated genes, red circles denote up-regulated circRNA, and dark green rhombuses represent down-regulated circRNA.

According to the prediction analysis with WebGestalt, 229 miRNA–DEmRNAs pairs were obtained to construct the miRNA–DEmRNA network, which contained 94 DEmRNAs and 20 miRNAs (Figure 2A). Among these, the top five miRNAs that had more co-expressive relationship with DEmRNAs included hsa-miR-200a-3p, hsa-miR-141-3p, hsa-miR-135b-5p, hsa-miR-135a-5p, and hsa-miR-182-5p. Moreover, the top five DEmRNAs with higher degrees in this network included *OGT*, *ZNF385A*, *BCL11A*, *MTSS1*, and *ATP1B1* (Table 1).

**Table 1:** The top 5 DEGs and miRNAs in miRNA-DEG regulatory network.

Genes	Description	Degree	miRNAs	Degree
*OGT*	down	12	hsa-miR-200a-3p	22
*ZNF385A*	up	8	hsa-miR-141-3p	22
*BCL11A*	up	8	hsa-miR-135b-5p	18
*MTSS1*	up	7	hsa-miR-135a-5p	18
*ATP1B1*	down	7	hsa-miR-182-5p	18

Moreover, we further used miRanda software to predict the combination of these 20 miRNAs and DEcircRNAs, total 57 miRNA–DEcircRNA regulatory interactions were selected, and miRNA–circRNA network included 42 DEcircRNAs and 17 miRNAs (Figure 2B). The top five miRNAs (hsa-miR-362-5p, hsa-miR-103a-3p, hsa-miR-526b-5p, hsa-miR-513a-5p, and hsa-miR-511-5p) and circRNAs (hsa_circ_023686, hsa_circ_045054, hsa_circ_001807, hsa_circ_006089, and hsa_circ_018993EPG5) identified based on the degree ranking are listed in Table 2.

**Table 2:** The top 5 DEcircRNAs and miRNAs in miRNA-DEcircRNA network.

circRNAs	Description	Degree	miRNAs	Degrees
hsa_circ_023686	up	3	hsa-miR-362-5p	6
hsa_circ_045054	up	3	hsa-miR-103a-3p	6
hsa_circ_001807	up	3	hsa-miR-526b-5p	6
hsa_circ_006089	up	3	hsa-miR-513a-5p	5
hsa_circ_018993	up	2	hsa-miR-511-5p	5

### Construction of ceRNA network

**Figure 3:**
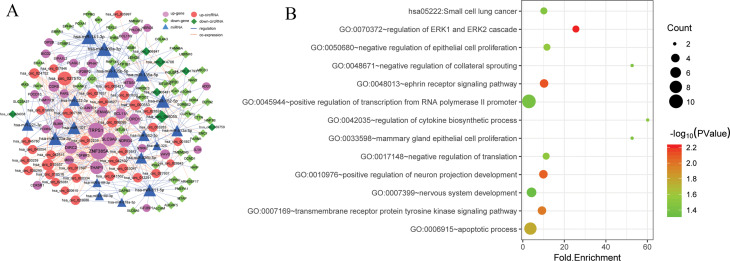
The ceRNA regulatory network and functional enrichment analysis of DEmRNAs in ceRNA network. (A) The ceRNA network. Pink circles represent up-regulated genes, blue triangles represent miRNAs, light green rhombuses indicate up-regulated cicrRNAs, deep green rhombuses represent down-regulated cicrRNAs, blue solid lines depict the regulatory relations among different RNA transcripts, and dotted orange line indicates the co-expression relationships. (B) Functional enrichment analysis of DEmiRNAs in ceRNA network. The dot size shows the gene ratio and color ranging from blue to red indicates the increasing significance.

To investigate the ceRNA regulation in LUSC and LUAD, the miRNA–DEmRNA pairs and miRNA–DEcircRNA pairs were integrated to establish the ceRNA network. The ceRNA network comprised 86 DEmRNAs, 42 DEcircRNAs, and 17 miRNAs (Figure 3A). Top 5 DEmRNAs, DEcircRNAs, and miRNAs are shown in Table 3. Additionally, the functional enrichment analysis of DEmRNAs in ceRNA network was performed. As showed in Figure 3B and Table 4, GO analysis revealed that DEGs were significantly enriched in the regulation of *ERK1* and *ERK2* cascade (GO:0070372), transmembrane receptor protein tyrosine kinase signaling pathway (GO:0007169), and negative regulation of epithelial cell proliferation (GO:0050680). These genes, including *EPHA2*, *EPHA7*, *NTRK2*, and *CDK6*, had different patterns in two major histological subtypes of NSCLC.

**Table 3:** The top 5 DEGs, DEcircRNAs, and miRNAs in ceRNA network.

DEGs	Description	Degree	DEcircRNAs	Description	Degree	miRNAs	Degree
ZNF385A	up	33	hsa_circ_027570	up	23	hsa-miR-141-3p	26
TRPS1	up	29	hsa_circ_010557	up	15	hsa-miR-200a-3p	24
SLC9A9	up	21	hsa_circ_000553	up	12	hsa-miR-182-5p	21
THAP1	up	19	hsa_circ_006089	up	10	hsa-miR-103a-3p	20
DIRC2	up	17	hsa_circ_014706	down	10	hsa-miR-135a-5p	20

**Table 4:** The functional enrichment analysis of DEmRNAs in ceRNA network.

Category	Term	Count	P Value	Genes
KEGG	hsa05222:Small cell lung cancer	3	0.034643	*COL4A3, CCND1, CDK6*
GO_BP	GO:0070372~regulation of ERK1 and ERK2 cascade	3	0.006112	*EPHA7, TGFBR3, EPHA2*
GO_BP	GO:0048013~ephrin receptor signaling pathway	4	0.007751	*EPHA7, CDK5R1, VAV3, EPHA2*
GO_BP	GO:0010976~positive regulation of neuron projection development	4	0.008515	*NDRG4, BCL11A, ENC1, NTRK2*
GO_BP	GO:0007169~transmembrane receptor protein tyrosine kinase signaling pathway	4	0.010466	*MTSS1, DOK4, PTPRG, NTRK2*
GOT_BP	GO:0006915~apoptotic process	8	0.017216	*CLPTM1L, SEMA6A, EVA1A, EPHA7, OGT, CAPN3, ZNF385A, IL1A*
GO_BP	GO:0050680~negative regulation of epithelial cell proliferation	3	0.02857	*MTSS1, TGFBR3, CDK6*
GO_BP	GO:0017148~negative regulation of translation	3	0.030483	*ENC1, IGF2BP2, IGF2BP3*
GO_BP	GO:0042035~regulation of cytokine biosynthetic process	2	0.032477	*IGF2BP2, IGF2BP3*
GO_BP	GO:0048671~negative regulation of collateral sprouting	2	0.037031	*EPHA7, BCL11A*
GO_BP	GO:0033598~mammary gland epithelial cell proliferation	2	0.037031	*CCND1, EPHA2*
GO_BP	GO:0045944~positive regulation of transcription from RNA polymerase II promoter	10	0.040381	*HNF1B, THRB, BCL11B, POU2F3, TRPS1, FOXA1, BCL11A, OGT, IL1A, PLAGL2*
GO_BP	GO:0007399~nervous system development	5	0.046348	*SEMA6A, DOK4, NAV2, ENC1, ATOH8*

### Survival analysis of DEmRNA

**Figure 4:**
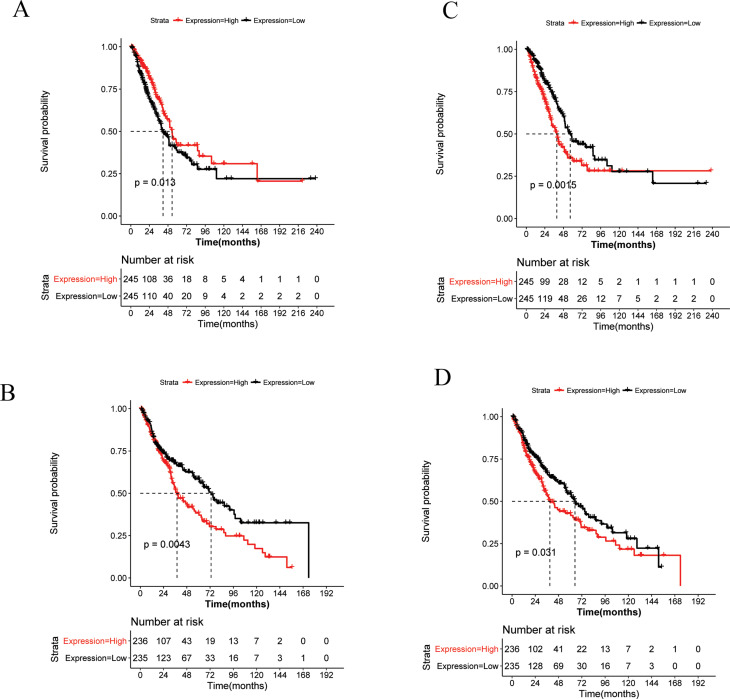
The overall survival curve of key genes. (A) *STXBP1* for LUAD, (B) *PMEPA1* for LUAD, (C) *STXBP1* for LUSC, and (D) *PMEPA1* for LUSC.

**Figure 5:**
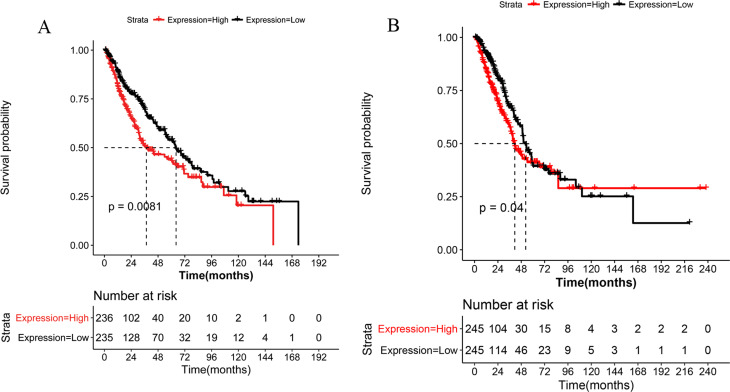
The survival curve of *EPHA2* (A) and *CDK6* (B). Red line represents high risk group and black line denotes low risk group.

Based on the expression profile data and clinical information of TCGA-LUAD and TCGA-LUSC, the survival analysis of 86 DEmRNAs in ceRNA network was performed. Among these, 22 genes exhibited remarkable correlation with the prognosis of LUAD, whereas 9 genes were closely associated with LUSC clinical outcomes (Table S4). Interestingly, both *STXBP1* and *PMEPA1* were significantly related to the prognosis of LUAD and LUSC. Furthermore, decreased expression of *STXBP1* predicted a relatively worse prognosis for LUAD (Figure 4A), whereas elevated *STXBP1* expression level represented a poor LUSC clinical outcome (Figure 4C). Additionally, the overexpression of *PMEPA1* was associated with LUAD and LUSC inferior prognosis (Figure 4B and 4D). We also noted that a high expression level of *EPHA2* represented a poor prognosis for LUSC (Figure 5A) and high level of *CDK6* indicated a poor prognosis for LUAD (Figure 5B).

## Discussion

In this study, we performed a systematic bioinformatics analysis to compare the gene expression patterns of LUSC and LUAD. A total of 635 DEmRNAs and 245 DEcircRNAs were identified in the LUSC vs. LUAD group. The ceRNA analysis identified several key nodes with different patterns in LUAD and LUSC, such as *EPHA2*, *EPHA7*, *NTRK2*, *CDK6*, hsa-circ_035997, hsa_circ_027570, and hsa_circ_006089. Additionally, functional enrichment analysis revealed that these genes were significantly enriched in the regulation of *ERK1* and *ERK2* cascade, transmembrane receptor protein tyrosine kinase signaling pathway, and negative regulation of epithelial cell proliferation pathway. Finally, we found that *STXBP1* and *PMEPA1* could be prognostic factors for both LUAD and LUSC, whereas *EPHA2* and *CDK6* could be diagnostic marker for LUSC and LUAD, respectively.

Eph receptor A7 (*EPHA7*) belongs to the receptor subfamily of the protein-tyrosine kinase family, which inhibits cancer survival and migration via a ligand and tyrosine kinase dependent signaling (Oricchio *et al.*, 2011). Giaginis *et al.* (2014) indicated that *EPHA7* was up-regulated in lung cancer tissues and positively associated with the proliferation of lung cancer cells. Subsequently, Liu *et al.* (2016) confirmed that knockdown of *EPHA7* could suppress the growth of NSCLC cells, suggesting that silencing *EPHA7* might provide a novel approach for the treatment of NSCLC. Moreover, the functional enrichment analysis implied that *EPHA7* was significantly enriched in the regulation of *ERK1* and *ERK2* cascade. *ERK1*/*ERK2* signaling cascades have been widely reported to serve vital roles in various diseases, such as cancer and chronic inflammation (Lu and Malemud, 2019). Lim *et al.* (2017) indicated that coumestrol (a novel chemotherapeutic agent) might play a therapeutic role in prostate cancer by mediating *ERK1/2* signaling pathway. Thus, we speculated that *EPHA7* played an important role in NSCLC via affecting the regulation of ERK1 and ERK2 cascade. In this study, we observed that the expression level of *EPHA7* was different in LUSC and LUAD. *EPHA7* expression was significantly correlated with the overall survival (OS) of LUAD patients and not with the OS of patients with LUSC (Giaginis *et al.*, 2014), indicating that the secretory form of *EPHA7* might have the potential to distinguish the type of tumor histopathology in lung cancer. However, further research based on large cohorts need to be performed to explore the function of *EPHA7* in LUAD and LUSC.

Another gene, neurotrophic receptor tyrosine kinase 2 (*NTRK2*), also presented different expression level in LUAD and LUSC. *NTRK2* encodes a member of the neurotrophic tyrosine kinase (*NTRK*) family, which phosphorylates itself and the members of the MAPK pathway by binding to neurotrophins (Park *et al.*, 2017). Gomez *et al.* (2018) compared potential therapeutic targets in LUSC and LUAD using proteomic analysis, they observed that the expression level of *NTRK2* was significantly increased in LUSC as compared to LUAD, and the combination of *NTRK2* with tyrosine kinase inhibitors could be considered as a promising approach for NSCLC treatment. Similarly, NTRK2 was found highly specific in LUSC (96.4%) compared with other carcinoma subtypes (including LUAD), suggesting that *NTRK2* was a potential immunohistochemical marker that might be particularly helpful in separating LUSC from LUAD (Terry *et al.*, 2011). Therefore, *NTRK2* might be a target for personalized treatment of NSCLC.

In addition to mRNA, our study also revealed that several circRNAs showed distinct expression patterns between LUSC and LUAD, such as hsa_circ_027570, hsa_circ_006089, and hsa-circ_035997. Unfortunately, the biological function of these circRNA in the pathogenesis of NSCLC has not been reported. The ceRNA analysis indicated that hsa_circ_006089 and hsa_circ_027570 bound to hsa-miR-103a-3p. Fan *et al.* (2019) confirmed that miR-103a-3p played an anti-oncogenic role in cancer, suggesting that it might serve a novel potential therapeutic target for NSCLC. Moreover, hsa-circ_035997 was bound to miR-141-3p. The expression level of miR-141-3p was significantly down-regulated in NSCLC tissues, suggesting that miR-141-3p might be a prognostic tumor suppressor involved in the NSCLC progression (Li *et al.*, 2019). Thus, we speculated that these circRNA might play roles by targeting miRNAs. However, the roles of these circRNAs in distinguishing LUAD from LUSC need further investigate.

Notably, our survival analyses suggested that two down-regulated genes (*STXBP1* and *PMEPA1*) were responsible for both LUAD and LUSC. Syntaxin binding protein 1 (*STXBP1*) encodes a syntaxin-binding protein, which plays a role in release of neurotransmitters via regulation of syntaxin (a transmembrane attachment protein receptor) (Rezazadeh *et al.*, 2019). A recent article reported the relationship between *STXBP1* and prognosis of LUAD (Wang *et al.*, 2020). They observed that *STXBP1* expression phenotypes could be categorized as membrane, cytoplasm, and mixed expression (both membrane and cytoplasm), revealing that entire *STXBP1* phenotypes or membrane phenotypes were closely connected with poor prognosis of LUAD and considered as independent prognostic factors of LUAD. Additionally, the detection of *STXBP1* helped in screening the patients with poor prognosis and more accurately strengthen adjuvant therapy. Consistent with previous study, our study also revealed the correlation between *STXBP1* expression and LUAD prognosis. However, we did not considered the relationship between STXBP1's subcellular localization and LUAD prognosis, and this will would be a focus of our further research. Notably, we also observed an association between *STXBP1* and LUSC prognosis, whereas the expression pattern of *STXBP1* in LUSC was different from that in LUAD. Thus, the function of *STXBP1* and its role in LUSC still need to be clarified. Prostate transmembrane protein, androgen induced 1 (*PMEPA1*) encodes transmembrane protein containing the Smad interaction motif (SIM), which could inhibit the transforming growth factor signaling pathways and play a role in multiple types of cancer (Fournier *et al.*, 2015; Zhang *et al.*, 2019). *PMEPA1* was found highly expressed in the lung cancer cell lines, whereas knockdown of *PMEPA1* could significantly inhibit the proliferation of cancer cells (Vo Nguyen *et al.*, 2014). Overexpression of *PMEPA1* was associated with poor prognosis of lung cancer, which was in line with our findings. Taken together, *STXBP1* and *PMEPA1* might be prognostic and diagnostic markers of NSCLC.

We observed that *EPHA2* was a prognostic factor for LUSC. *EPHA2* mutation was present in LUSC, and it could increase tumor invasion and survival by activating the focal adhesions and actin cytoskeletal regulatory proteins, indicating *EPHA2* as a potential therapeutic target of LUSC (Faoro *et al.*, 2010), which was consistent with our findings. In addition, the expression level of *CDK6* was significantly associated with the prognosis of LUAD. Cyclin-dependent kinase 6 (*CDK6*) was reported to be a vital regulator of cell cycle progression and correlated with various cancers including NSCLC. The expression level of CDK6 was negatively associated with the overall survival of patients with LUAD (Li *et al.*, 2017), which was further confirmed in our results. Taken together, we speculated that *EPHA2* and *CDK6* were potential biomarkers in the personalized treatment for LUSC and LUAD, respectively.

There were some limitations in our study. Our results only indicated that the obtained DEmRNAs and DEcircRNAs had different expression patterns in the LUSC and LUAD, whether they served roles in the development of NSCLC needs to be explored. In addition, our study was performed based on bioinformatics analysis, further experimental studies were demanded to validate our results.

In summary, we compared two major histological subtypes of NSCLC (LUAD and LUSC) based on mRNA and circRNA expression patterns. We identified several genes, such as *EPHA2*, *EPHA7*, *NTRK2*, *CDK6*, hsa_circ_027570, and hsa_circ_035997, might be used to distinguish LUAD and LUSC. Notably, *STXBP1* and *PMEPA1* could predict clinical outcomes of both LUAD and LUSC. Meanwhile, *EPHA2* and *CDK6* might be considered as prognostic biomarkers for LUSC and LUAD, respectively.

## Supplementary material

The following online material is available for this article:

Table S1 - The clinical characteristics of included patients.

Table S2 - The list of differentially expressed mRNAs between LUSC and LUAD.

Table S3 - The list of differentially expressed circRNAs between LUSC and LUAD.

Table S4 - The differentially expressed genes related to prognosis.
