# Supplementary material for: A comparison of mRNA and circRNA expression between squamous cell carcinoma and adenocarcinoma of the lungs
Source: Genet Mol Biol. 2020 Nov 6;43(4):e20200054. doi: 10.1590/1678-4685-GMB-2020-0054 (PMC7654371; doi:10.1590/1678-4685-GMB-2020-0054)
Supplement: Supplementary file 1 [file 1415-4757-GMB-43-4-e20200054-s1.pdf]

Genetics and Molecular Biology

https://doi.org/10.1590/1678-4685-GMB-2020-0054

Supplementary Material to "A comparison of mRNA and circRNA expression between squamous cell carcinoma and adenocarcinoma of the lungs."

Table S1 - The clinical characteristics of included patients

Type	NO.	Gender	Age	Smoking history	TNM	Stage	Adjuvant therapy
LUSC	1	F	71	None	PT3N2M0	IIIA	NO
LUAD	2	M	69	None	pT3N1M1	IVB	NO
LUSC	3	M	50	20 years (20/day)	pT2N0M0	IB	NO
LUSC	4	M	51	30 years (20/day)	PT3N2M0	IIIA	NO
LUAD	5	F	71	<400/year	T2aN0M0	Ib	NO
LUSC	6	M	67	None	T3N2M0	IIIA	NO
LUAD	7	M	69	A little	pT2aN0M0	Ib	NO
LUSC	8	F	57	30 years (20/day)	T3N1M0	IIIA	NO
LUAD	9	M	69	30 years (30/day)	pT2N2M0	IIIA	NO
LUAD	10	F	54	None	pT2aN1M0	IB	NO
LUAD	11	F	55	None	pT2N0M0	IB	NO

Type	NO.	Gender	Age	Smoking history	TNM	Stage	Adjuvant therapy
LUAD	12	F	58	None	pT2N1M0	IIB	NO
LUAD	13	M	68	20 years (20/day)	pT2N0M0	IB	NO
LUAD	14	M	60	30 years (10/day)	pT3N2M0	IIIA	NO
LUAD	15	F	61	40 years (40/day)	pT3N1M0	IIB	NO
LUAD	16	M	65	40 years (20/day)	pT3N2M0	IIIA	NO
LUAD	17	F	73	None	pT3N2M0	IIIA	NO
LUSC	18	M	60	20years (20/day)	pT3N2M0	IIIA	NO
LUSC	19	M	61	30years (10/day)	pT3N2M0	IIIA	NO
LUSC	20	F	70	None	pT3N1M0	IIB	NO
