# Supplementary material for: A comparison of mRNA and circRNA expression between squamous cell carcinoma and adenocarcinoma of the lungs
Source: Genet Mol Biol. 2020 Nov 6;43(4):e20200054. doi: 10.1590/1678-4685-GMB-2020-0054 (PMC7654371; doi:10.1590/1678-4685-GMB-2020-0054)
Supplement: Supplementary file 4 [file 1415-4757-GMB-43-4-e20200054-s4.pdf]

*Genetics and Molecular Biology*

https://doi.org/10.1590/1678-4685-GMB-2020-0054

# Supplementary Material to "A comparison of mRNA and circRNA expression between squamous cell carcinoma and adenocarcinoma of the lungs."

**Table S4** - The differentially expressed genes related to prognosis

| LUSC | | LUAD | |
|---|---|---|---|
| Gene | Pvalue | Gene | Pvalue |
| **STXBP1** | 0.00428695 | IRX5 | 1.55E-05 |
| EPHA2 | 0.00813035 | MTURN | 0.0002359 |
| ADD3 | 0.01073184 | OGT | 0.0004993 |
| SLC9A9 | 0.01099923 | **PMEPA1** | 0.0015137 |
| EVA1A | 0.01130279 | IGF2BP3 | 0.0020889 |
| SHANK2 | 0.0119727 | DCUN1D1 | 0.0052575 |
| FAM46A | 0.02016478 | DIP2B | 0.0077857 |
| **PMEPA1** | 0.03070171 | **STXBP1** | 0.0129428 |
| NAV2 | 0.04797483 | TMEM243 | 0.0143325 |
| | | IGF2BP2 | 0.0204467 |
| | | WFDC2 | 0.0207173 |
| | | CAPN3 | 0.0269737 |
| | | PARM1 | 0.0317552 |
| | | PDCD10 | 0.0345579 |
| | | PARL | 0.0355587 |
| | | ARRDC3 | 0.0365931 |
| | | CDK6 | 0.0396845 |
| | | DOK4 | 0.0408639 |

| LUSC | LUAD | |
|---|---|---|
| | CORO1C | 0.0437517 |
| | WSB1 | 0.0444235 |
| | ERRFI1 | 0.0461206 |
| | PTPRG | 0.048331 |

The bold part is the prognostic genes associated with both LUSC and LUAD.
